# Grateful Patients

**Published:** 1887-05-28

**Authors:** 


### The Editor's Letter-Box.

[Our Correspondents are reminded that prolixity is a great bar to publication, and that brevity of style and conciseness of statement greatly facilitate early publication.]

### GRATEFUL PATIENTS.

The Secretary of the Hospital for Epilepsy and Paralysis, Regent's Park, writes to us on this subject, saying that he has just received a cheque for five guineas from a former out-patient, who had already sent £5, and whose circumstances have now enabled her to express her gratitude in this tangible and welcome form.

